# Surface electroencephalography (EEG) during the acute phase of stroke to assist with diagnosis and prediction of prognosis: a scoping review

**DOI:** 10.1186/s12873-022-00585-w

**Published:** 2022-02-28

**Authors:** Lou Sutcliffe, Hannah Lumley, Lisa Shaw, Richard Francis, Christopher I. Price

**Affiliations:** Stroke Research Group, Population Health Science Institute, Newcastle University, Newcastle-Upon-Tyne, UK

**Keywords:** Electroencephalography, Acute stroke, Diagnosis, Prognosis, Large vessel occlusion

## Abstract

**Background:**

Stroke is a common medical emergency responsible for significant mortality and disability. Early identification improves outcomes by promoting access to time-critical treatments such as thrombectomy for large vessel occlusion (LVO), whilst accurate prognosis could inform many acute management decisions. Surface electroencephalography (EEG) shows promise for stroke identification and outcome prediction, but evaluations have varied in technology, setting, population and purpose. This scoping review aimed to summarise published literature addressing the following questions: 1. Can EEG during acute clinical assessment identify: a) Stroke versus non-stroke mimic conditions. b) Ischaemic versus haemorrhagic stroke. c) Ischaemic stroke due to LVO. 2. Can these states be identified if EEG is applied < 6 h since onset. 3. Does EEG during acute assessment predict clinical recovery following confirmed stroke.

**Methods:**

We performed a systematic search of five bibliographic databases ending 19/10/2020. Two reviewers assessed eligibility of articles describing diagnostic and/or prognostic EEG application < 72 h since suspected or confirmed stroke.

**Results:**

From 5892 abstracts, 210 full text articles were screened and 39 retained. Studies were small and heterogeneous. Amongst 21 reports of diagnostic data, consistent associations were reported between stroke, greater delta power, reduced alpha/beta power, corresponding ratios and greater brain asymmetry. When reported, the area under the curve (AUC) was at least good (0.81–1.00). Only one study combined clinical and EEG data (AUC 0.88). There was little data found describing whether EEG could identify ischaemic versus haemorrhagic stroke. Radiological changes suggestive of LVO were also associated with increased slow and decreased fast waves. The only study with angiographic proof of LVO reported AUC 0.86 for detection < 24 h since onset. Amongst 26 reports of prognostic data, increased slow and reduced fast wave EEG changes were associated with future dependency, neurological impairment, mortality and poor cognition, but there was little evidence that EEG enhanced outcome prediction relative to clinical and/or radiological variables. Only one study focussed solely on patients < 6 h since onset for predicting neurological prognosis post-thrombolysis, with more favourable outcomes associated with greater hemispheric symmetry and a greater ratio of fast to slow waves.

**Conclusions:**

Although studies report important associations with EEG biomarkers, further technological development and adequately powered real-world studies are required before recommendations can be made regarding application during acute stroke assessment.

**Supplementary Information:**

The online version contains supplementary material available at 10.1186/s12873-022-00585-w.

## Background

Stroke is responsible for a high disability, mortality and economic burden worldwide. Emergency treatments can improve outcomes [1, 2], particularly intravenous thrombolysis and mechanical thrombectomy for selected patients with ischaemic stroke. These highly time-sensitive treatments reduce long-term disability when administered to appropriate patients, but urgent clinical assessment including brain Computed Tomography (CT) or Magnetic Resonance Imaging (MRI) must first determine eligibility. For mechanical thrombectomy, additional angiography (CTA or MRA) is needed to confirm the presence of large vessel occlusion (LVO), with subsequent transfer of treatable patients if they are not already at a comprehensive stroke centre [3]. Earlier identification of individual patients most likely to benefit from specific emergency treatments will improve outcomes, especially if this is possible in the prehospital setting so that ambulance admissions can be directed to the most appropriate facility.

Accurate initial identification of stroke patients is complicated by ‘mimic’ conditions that produce the same symptoms as stroke, such as epileptic seizures, migraine and infections. A literature review of 79 studies reported that, despite routine use of symptom checklists like the Face Arm Speech Test, an average of 27% (range: 4–43%) prehospital suspected stroke admissions and 10% (range: 1–25%) thrombolysis patients were later re-categorised as stroke mimics [4]. More complex symptom checklists have been developed to identify LVO, but these have not been widely adopted due to the unfavourable balance between specificity and sensitivity [5, 6]. Point-of-care tests to distinguish stroke from mimic patients, haemorrhagic from ischaemic stroke and/or identify LVO would allow earlier access to appropriate emergency care. Although prehospital CT brain imaging in mobile stroke units has been implemented within highly resourced healthcare systems [7], there are no other diagnostic technologies currently available with acceptable accuracy [8]. Similarly, portable technologies providing early information about prognosis could assist clinicians whilst making a range of acute management decisions, such as whether treatment of early complications would be likely to influence recovery or might possibly be futile.

Electroencephalography (EEG) is a non-invasive clinical tool frequently used in hospital-based diagnosis and management of seizures, but has also been evaluated for stroke identification and prognostication. An increase in slow-wave (delta) versus faster (alpha/beta) activity has long been recognised following a recent stroke, although the exact mechanism is uncertain [9–11]. Quantitative EEG (qEEG) has been used as a biomarker to predict outcomes in ischaemic stroke in acute and sub-acute settings [12, 13]. Its ability to detect and size lesions [14, 15] suggests that it could be used as a diagnostic tool and a clinical decision aid during treatment decisions. Advances in qEEG analysis methods and algorithms such as the Brain Symmetry Index [16], and introduction of portable systems using a minimal number of electrodes [17, 18], have increased the practical potential for use in emergency department (ED) and pre-hospital settings [19]. We undertook a literature review to describe the use of EEG during the acute phase of stroke for stratification of unselected patients into important clinical groups, and as an aid for clinical decision-making through early estimation of prognosis. A scoping review approach was applied due to significant heterogeneity in technology and setting in this emerging field.

## Methods

The Preferred Reporting Systems for Systematic Reviews and Meta-Analyses Extension for Scoping Reviews (PRISMA-ScR) framework was applied [20].

## Aim

The aim was to report evidence describing the capability of EEG technologies for stratification (identification and prognostication) when applied within 72 h of stroke symptom onset.

## Objectives

By classifying and describing clinical studies of EEG technologies applied soon after stroke symptom onset (< 72 h), we addressed the following questions:

1. Can EEG during acute clinical assessment identify:
  - Stroke versus non-stroke mimic conditions.
  - Ischaemic versus haemorrhagic stroke.
  - Ischaemic stroke due to LVO.
2. Can these states be identified if EEG is applied < 6 h of symptom onset.
3. Does EEG during acute assessment predict clinical recovery following confirmed stroke.

### Search strategy

Following exploratory searches, a systematic strategy combining MeSH/Web of Science categories and keywords was developed and executed in Ovid (selecting Medline, Embase and PsycINFO databases), Web of Science and Scopus databases up until the 19^th^ October 2020 inclusive. Hand searching of reference lists and citation searches of included studies were undertaken. Only published peer-reviewed literature was retained, including conference abstracts if there was sufficient information reported, but case studies were excluded. It was not necessary to contact the authors of any articles for clarification. The search strategies are listed under ‘Supplement A’ in the Supplementary Material.

### Study inclusion criteria

Research studies and review articles, including feasibility and pilot studies, with abstracts published in English from any country were eligible for inclusion if they presented original data and appropriate statistical comparison describing the application of EEG technology for stroke identification or prognosis. It was necessary for the test population to include patients with suspected or confirmed stroke, where the EEG technique was commenced (but not necessarily completed) within 72 h. Although this time window extended beyond the interval for delivery of emergency stroke treatments, it enabled inclusion of information from studies with a range of onset to EEG times. Studies that focused mainly or solely on seizures (including prediction of post-stroke epilepsy) or Transient Ischaemic Attack (TIA) (stroke symptoms resolved within 24 h) were excluded.

Any EEG-based assessment was permissible, including but not limited to: qualitative visual analysis of EEG, qEEG, continuous EEG monitoring, the Brain Symmetry Index (BSI) and frequency-specific power measures such as delta/alpha power ratio (DAR) or (delta + theta)/(alpha + beta) power ratio (DTABR). The study setting could be in hospital or in an ambulance, including situations where patients were conveyed to a specialist laboratory from hospital for EEG recording.

Any diagnostic process was accepted for the stroke reference standard i.e. MRI/A, CT/A and/or specialist opinion. Comparisons against mimic conditions and non-stroke/healthy controls were included when the origin of the source data was stated. However, studies were not included if stroke patient data were being compared only to standard definitions of ‘healthy/normal’ EEG parameters, without description of a reference data source.

Studies examining detection of LVO were included if there was direct evidence of large artery occlusion (e.g. CT angiography) or, because not many studies were expected to use this reference standard, we also considered studies reporting indirectly associated radiological features (e.g. large infarct size).

For prognostic studies we included those using any previously described clinical stroke outcome measure, or survival/death. For these studies, we reported only the main outcome of interest as stated by the authors.

### Study selection

Duplicate articles were excluded. Two members of the study team (LSu + RF) reviewed titles and abstracts and selected full text articles to confirm inclusion with arbitration by a third reviewer if required (CP and/or LSh). Templates for review, extraction and quality assessment can be found under ‘Supplement B’ in the Supplementary Material.

### Data extraction

Data were independently extracted by two reviewers (LSu and HL), with discrepancies resolved via group discussion.

A data extraction framework was developed and piloted by the reviewers before use, which included fields for: Year of publication, country of origin, study aims, study design, setting, inclusion/exclusion criteria, EEG technology, EEG data processing methodology, reference standard information, outcome measures, blinding, sample size, time from stroke onset to first EEG measure, major findings (including statistical significance and diagnostic accuracy) and whether all patients were represented in the data with any exclusions explained.

To assess study quality, a simple scoring system (0–5) was created which reflected the main indicators of good research design i.e. clear eligibility criteria; clearly defined technology; clearly defined reference standard and/or outcome measure; blinding; whether all participants were accounted for in the results presented. Studies were not excluded based on quality, but quality and design were considered during recommendations based upon strength of evidence.

### Data synthesis

As this was a scoping review, there was no a-priori plan for data meta-analysis and a narrative description is provided. Data are presented in tables according to reference standard or outcome measure in ascending order of publication date.

## Results

**Fig. 1:**
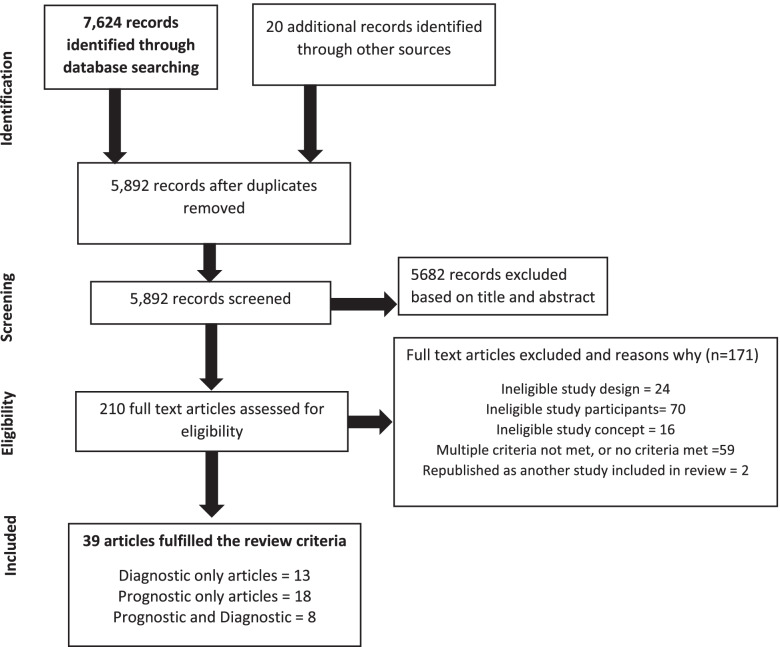
Flow diagram summarising the process used to identify studies

Databases searches identified 7624 articles, with 20 more from hand-searching relevant review publications. After removal of duplicates, 5892 abstracts remained. Of these, 5682 abstracts did not meet the inclusion criteria. The remaining 210 full text articles were assessed (Fig. 1) and 171 articles were excluded: 24 did not meet study design criterion, 70 did not meet the participants criterion, 16 did not address the review question, 59 did not meet multiple criteria, and 2 were republished as another included study. After full text review, 39 articles were included for data extraction and quality assessment: 13 reporting diagnostic data only, 18 reporting prognostic data only and eight articles reporting both.

### Included studies

Study designs were diverse. The majority were cohort (*n* = 24) or case–control (*n* = 14) studies, although very few specifically used these terms. Only one study [21] was considered a true diagnostic accuracy study, as the investigators performing the EEG were blinded to patients’ clinical status and the reference standard was determined in advance (clinical specialist opinion).

### Population

There was a wide geographic distribution of studies: Eight in China; seven in Australia; five in USA; three each in Belgium and Cuba; two each in Portugal and Israel and one each in Germany, Indonesia, Ukraine, Italy, Brazil, Finland, France and Hungary. Nationality was unclear for one conference abstract. Apart from one study where the setting was unclear, most were conducted in acute care settings in hospital (two in Emergency Departments; four in an Intensive Care Unit; seven in a neurology department; 10 in a stroke unit and 15 in hospital with no clear department). No studies were conducted in an ambulance or in the pre-hospital setting.

The median number of patients across the 39 articles was 33 (range = 11–199). Inclusion and exclusion criteria were extremely variable, with some studies requiring extensive lists of exclusions and others giving limited or no information beyond a diagnosis of “stroke”. Two diagnostic studies [22, 23] included TIA as part of the stroke patient sample, whereas others excluded TIA. Inclusion/exclusion criteria that appeared frequently are listed in Supplement C (Table S1) in the Supplementary Material.

Median time from stroke onset to EEG application was 48 h (range = 4.5–72 h) when this information was available. There was only one study where all patients were within six hours of symptoms onset, which was examining EEG indicators for recovery of neurological impairment after thrombolysis [24].

### EEG techniques

Most studies used quantitative EEG measures as their stroke biomarkers (*n* = 30). A smaller number used either topographic EEG mapping (*n* = 4) or qualitative analysis of abnormal EEG patterns such as epileptiform activity based on expert assessment (*n* = 13). Eight studies used multiple EEG markers. A single study made use of a deep learning neural network to select optimal diagnostic EEG conditions [23]. All studies that reported electrode locations used the international 10/20 system (*n* = 36). Characteristics of EEG techniques commonly specified by included studies are listed in Supplement C (Table S2) in the Supplementary Material.

### Reference standards and outcome measures

Amongst 21 articles reporting diagnostic information, the most common reference standard was “specialist opinion including imaging” (*n* = 11). Five articles used ‘specialist opinion’ without providing further detail. Five articles used CT or MRI, one study used CT and one study used MRI alone. In 12 studies the reference standard assessment was performed within 72 h or during the inpatient stay. In three more, it was additionally recorded over a longer time-window (up to 118 h), and in six there was no time reported.

Amongst 26 studies reporting prognostic information, the most frequent outcome measure was an assessment of dependency (*n* = 12) including modified Rankin Score (mRS), Glasgow Outcome Scale (GOS), and Barthel Index (BI). Eight studies assessed neurological outcome using the National Institutes of Health Stroke Scale (NIHSS). Smaller numbers of articles assessed cognitive impairment via the Montreal Cognitive Assessment (MoCA) or a dementia diagnosis (*n* = 4), and survival/mortality (*n* = 2). Most studies assessed the outcome measure after discharge or more than 72 h after stroke, at a time point ranging from seven days to seven years.

### Quality of studies

Only two articles showed evidence of a sample size calculation [25, 26]. One other article included a post-hoc power calculation and ascertained that only some of their EEG parameters/sub-analyses had adequate statistical power [15].

Sixteen articles had some evidence of outcome blinding. It was stated that the EEG assessor was blinded to clinical data in the reference standard for three diagnostic articles, but there was no explicit indication that the clinician assessing the reference standard was blinded to EEG data. Eight articles with a prognostic aim reported a variety of blinding methods: EEG and outcome assessors blinded (*n* = 5), only EEG assessor blinded (*n* = 3), only outcome assessor blinded (*n* = 1) and patients blinded (*n* = 1). For three articles that had both diagnostic and prognostic aims and any form of blinding, there was evidence that the EEG assessor (or secondary EEG assessor) or outcome assessor was blinded to clinical data.

There were five articles where it was not possible to account for all the participants due to unclear text, figures, or presentation of data representing only individual patients.

Of the 21 articles with a diagnostic aim, 15 had evidence of a predetermined reference standard including specialist opinion. Of the 26 articles with a prognostic aim, the outcome measure was clearly defined for five studies but the majority were unclear as to whether a measure had been selected before commencing recruitment.

### Study data

#### 1a) Identification of stroke versus non-stroke

Seventeen articles considered whether EEG could distinguish stroke from non-stroke; two of which specifically aimed to distinguish between stroke and TIA. Studies are summarised in Table 1, grouped by year of publication and reference standard.

**Table 1:** EEG during acute clinical assessment to identify stroke versus non-stroke conditions

**Reference**	**Reference standard**	Participants	Key exclusions	First EEG start time after onset (h)	EEG procedure	EEG processing	EEG biomarker	Result	Quality score
**Cohen 1977 **[27]	**Specialist opinion based on ‘routine clinical assessment form’**	26 Ischaemic Stroke patients, 26 controls	Previous stroke	< 72	19 electrodes	Offline filter 0.35-35 Hz, 1 min epochs	Absolute spectral power	Stroke participants exhibited significant interhemispheric delta power asymmetry vs non-stroke (*p* < 0.05)	2
**Yan 2011 **[28]	**Specialist opinion**^b^	22 Stroke patients, 10 controls	Not Reported	< 48	16 electrodes, eyes closed, resting	Offline visual artifact removal followed by digital filter, 10 s epochs. FFT	BBSI	Higher BBSI in stroke vs non-stroke (diagnostic accuracy = 83% when conscious, 71.43% unconscious)	2
**Aminov 2017 **[25]	**Specialist opinion**^b^	15 Ischaemic Stroke patients, 4 Haemorrhagic Stroke patients, 19 controls (database)	History of neurological/ psychiatric disorders	< 72	Single electrode at FP1, eyes closed	Online filter 0.5-30 Hz, manual artifact removal, 4 s epochs; FFT	Relative spectral power (DAR, DTR)	Less theta power (*p* = 0.02), more delta (*p* < 0.01) power, higher DAR (*p* < 0.01) and DTR (*p* = 0.01) in stroke participants vs non-stroke	4
**Erani 2020 **[23]	**Specialist opinion**^b^	43 Ischaemic Stroke patients, 7 Haemorrhagic Stroke patients, 13 TIA patients, 37 Stroke mimics	Not Reported	< 23	17 electrodes, portable, dry electrode system, eyes open, resting	Offline analysis: filtering, noise removal and re-referencing. EEG variables selected using Lasso regression	Relative spectral power (all bands, beta split into low and high) Diagnostic neural network	Deep learning EEG (4 lasso selected electrode pairs) and clinical data model could identify stroke/TIA from mimic (AUC = 0.88, sensitivity = 79%, specificity = 80%) more accurately than combined clinical and EEG (4 electrode pairs) data (AUC = 0.80, sensitivity = 70%, specificity = 80%) and individual EEG (4 electrode pairs) (AUC = 0.78, sensitivity = 65%, specificity = 80%) or clinical (AUC = 0.62, sensitivity = 40%, specificity = 80%) data models. Less high frequencies (alpha and high beta 20.5-28 Hz) and greater low frequencies (low beta 12.5-16 Hz) associated with stroke/TIA	4
**Rodriguez 2012 **[29]	**Admission CT/** **Specialist opinion**	29 Ischaemic Stroke patients, 15 Haemorrhagic Stroke patients (all MCA), Unknown no. of controls (database)	Not Reported	< 72	Not Reported	Not Reported	Relative spectral power (all bands, DAR, PRI)	Significant increase in slow wave frequencies (< 6.25 Hz) and decrease in alpha/beta in stroke versus control. Significantly greater PRI and DAR in stroke patients vs non-stroke (abstract-no statistics given)	3
**Chen 2018 **[30]	**Specialist opinion informed by CT**	47 Haemorrhagic Stroke patients, 15 controls	Ruptured aneurysm; vascular malformation or stenosis; cerebral trauma; tumour; encephalitis; ischaemic stroke; previous stroke; CNS depressants	< 59	Controls eyes closed and awake	Offline filters > 0.3, < / = 30 Hz, artifacts removed. FFT	Relative spectral power delta, alpha, DAR, DTABR), BSI	Lower alpha power, greater delta power and higher DAR and DTABR in stroke patients vs non-stroke (all *p* < 0.0001). BSI was not significantly different	4
**Chan 2019 **[31]	**Specialist opinion informed by CT**	32 patients (Ischaemic Stroke and control; unclear division)	Haemorrhagic Stroke	< 72	32 electrodes, eyes open and closed, resting but conscious, hyperventilation and photic stimulation	Sampling 250 Hz and 512 Hz, FFT; DWT (Daubechies 4)	Relative spectral power (DAR, DTABR), BSI	Higher BSI, DAR, DTABR and greater delta power in stroke patients vs non-stroke. EEG identified stroke with > 87.5% accuracy	2
**Machado 2004 **[32]	**Specialist opinion based on CT, MRI, medical history and neurological exam**	32 Ischaemic (LMCA) Stroke patients 211 controls	Not Reported	< 24	19 electrodes	Online filters < / = 0.5, > 30, 60 Hz notch filter, sampling 200 Hz, EOG artifact removal, 2.56 s epochs	Tomography	Greater delta and theta and less alpha power in the territory of the stroke (all *p* < 0.01) compared to the same territory in non-stroke	3
**Finnigan 2016 **[33]	**Specialist opinion, based on CT/MRI within 6 h of onset**	18 Ischaemic (LMCA) Stroke patients, 28 controls	Non-cortical stroke; bilateral stroke; seizures; haemorrhage; previous neurological conditions; previous stroke; encephalitis	< 24	19 electrodes, eyes closed with checking for wakefulness	Sampling 500HZ, offline filter 0.5-40 Hz, 12 dB/octave,, EOG artifact removal, 2 s epochs	Relative spectral power (all bands, DAR, DTABR)	Greater delta (*p* < .0001, AUC = 0.99, sensitivity = 94%, specificity = 96%) and theta (*p* < .001, AUC = 0.81, sensitivity = 89%, specificity = 68%), lower alpha (*p* < .0001, AUC = 0.97, sensitivity = 89%, specificity = 93%) and beta (*p* < .0001, AUC = 0.9, sensitivity = 83%, specificity = 82%), higher DAR, (*p* < .0001, AUC = 1.0, sensitivity = 100%, specificity = 100%), DTABR (*p* < .0001, AUC = 0.99, sensitivity = 100%, specificity = 96%) and QSlowing (*p* < .0001, AUC = 0.97, sensitivity = 94%, specificity = 96%) in stroke vs non-stroke (*p* < .001)	3
**Rogers 2019 **[21]	**Specialist opinion based on CT, MRI, echocardiogram, bloods & ultrasound or CTA**	10 Ischaemic Stroke patients, 10 controls	History of neurological/ psychiatric disorders; current haemorrhagic stroke	< 72	Single electrode at FP1, Auditory Oddball EP, eyes closed and resting	Offline. filter 0.5-30 Hz, manual artifact removal	Relative spectral power (all bands)	Greater delta (AUC = 0.87, sensitivity = 90%, specificity = 85%) and less theta (AUC = 0.93, sensitivity = 85%, specificity = 90%) power in stroke vs control (both *p* = 0.03)	5
**Gottlibe 2020 **[34]	**Specialist opinion based on CT/MRI at baseline/admission**	33 Ischaemic Stroke patients, 25 controls	Degenerative neurological conditions; Seizure/epileptiform EEG	< 48	4 electrodes. Awake, alert, sitting position	Sampling 220 Hz, offline computer artifact removal, 10 min overlapping epochs, filter 0.16-76 Hz	r-BSI	Higher r-BSI in stroke vs non-stroke (*p* = 0.002)	3
**Finnigan 2020 **[35] ^a^	**Specialist opinion, based on CT/MRI within 6 h of onset**	18 Ischaemic Stroke (LMCA) patients, 28 controls	Non-cortical stroke; bilateral stroke; seizures; haemorrhage; previous neurological conditions; previous stroke; encephalitis	< 24	Six electrodes, eyes closed with checking for wakefulness	Offline filter 0.5-40 Hz, 12 dB/octave, EOG artifact removal, 2 s epochs	Relative spectral power (DAR)	Higher DAR stroke participants vs non-stroke using two frontal electrodes (F3-F4). AUC = 0.99, sensitivity = 93%, specificity = 94%	4
**Murri 1998 **[36]	**CT within 4 days of onset**	65 Ischaemic Stroke patients, 60 controls	Bilateral stroke; previous stroke; gradual onset; neurological or systemic pathologies	< 24	Eyes closed, supine with eye open breaks in a quiet, dimly lit room	Online filter 1-50 Hz, time constant 0.3 s, manual artifact removal, 4 s epochs	Topographic activity	Greater maximum delta power was observed in patients versus control subjects for cortical lesions: frontocentral *p* < 0.01, AUC = 0.68, sensitivity = 92%, specificity = 45%; Temporal *p* < 0.01, AUC = 0.85, sensitivity = 88%, specificity = 83%; Parieto-occipital *p* < 0.01, AUC = 0.75, sensitivity = 79%, specificity = 72%, (diagnostic accuracy extrapolated from true and false positive and negative values). Cortical lesions could be located using the electrode with maximum delta power (Kappa = 0.63 (0.39–0.87)) after striatocapsular lesions excluded. Amongst stroke patients conventional and topographic EEG had 73 and 84% sensitivity respectively for detecting focal lesions	4
**Luu 2001 **[37]	**CT or MRI**	6 Ischaemic Stroke patients, 16 controls	Haemorrhagic Stroke; Non-cortical Stroke; Previous stroke/other brain lesions; state altering or confounding medications; NIHSS < 8	< 36	Variable no of electrodes tested (19–128), eyes open and closed	Online filter 0.1-59 Hz, artifacts removed, 1 s epochs	Topographic activity	Increased slow wave (delta and theta) amplitude 2 standard deviations above mean in stroke related EEG versus control but only in 4/6 (67%) patients	5
**Shreve 2019 **[22]	**CT, MRI and NIHSS**	11 Ischaemic Stroke patients, 3 TIA patients, 10 mimic patients	Haemorrhagic Stroke	< 43.5	256 electrodes but 62 excluded, awake, fixed gaze with bed at 30 degree angle	Offline-only sixth order < 50 Hz filter, independent component analysis artifact removal, 1 s epochs	Relative spectral power (All bands, global power, DAR, DTABR)	No EEG measure significantly distinguished cerebral ischaemia from non-ischaemia	4
**Finnigan 2004 **[12]	**MRI (DWI) 15 h ± 3 h**	11 Ischaemic Stroke patients, 6 controls	Fever, encephalitis, seizures, ICH, non-cortical stroke, confounding neurological condition (e.g. previous stroke) or medication	< 9	64 electrodes, between MRI scans	Online filter .01-100 Hz, artifacts 0.2- 40 Hz, automatic artifact removal, 4 s epochs, sampling 500 Hz, FFT .5-50 Hz	Relative spectral power (aDCI)	Significantly greater mean delta power in patients versus controls (t = 4.68, *P* = 0.001). Control aDCI was at least 1 order of magnitude lower than the lowest patient aDCI	3
**Sheorajapanday 2009 **[26]	**MRI within 5 days**	21 Ischaemic Stroke patients, 10 controls	Not Reported	< 72	20 electrodes. Eyes closed, alert	Offline filter(s) > 0.3, < / = 30, manual artifact removal, FFT	Relative spectral power (all bands, DAR, DTAR, DTABR), pdBSI	pdBSI distinguished stroke from control patients (*p* = 0.0003; 1-25 Hz range *p* = 0.001) and correlated with clinical and radiological status (*P’s *< 0.001). No significant differences between groups for RAP, RDP, RDTP, DAR, DTAR or DTABR	3
**EEG to distinguish stroke from Transient Ischaemic Attack (TIA)**									
**Rogers 2019 **[21]	**Specialist opinion**	10 Ischaemic Stroke patients, 10 TIA patients	Neurological/ psychiatric disorders, SAH	< 72	Single electrode at FP1, Auditory Oddball EP, eyes closed and resting	Offline, filter 0.5-30 Hz, manual artifact removal	Relative spectral power (all bands)	Greater delta (AUC = 0.87, sensitivity = 90%, specificity = 85%) power in stroke vs TIA (*p* < 0.01). Greater alpha (AUC = 0.81, sensitivity = 80%, specificity = 90%) and beta (AUC = 0.86, sensitivity = 90%, specificity = 80%) power in TIA vs stroke (both *p* < 0.01)	5
**Sheorajapanday 2009 **[26]	**MRI within 5 days**	21 Ischaemic Stroke patients, 10 TIA patients	Not Reported	< 72	20 electrodes. Eyes closed, alert	Offline filter(s) > 0.3, < / = 30, manual artifact removal, FFT	Relative spectral power (all bands, DAR, DTAR, DTABR), pdBSI	pdBSI distinguished stroke from TIA patients (*p* = 0.0003; 1-25 Hz range *p* = 0.001). No significant differences between groups for RAP, RDP, RDTP, DAR, DTAR or DTABR	3

*RAP* Relative Alpha Power, *RDP* Relative Delta Power, *RDTP* Relative Delta and Theta Power, *Adci* Acute Delta Change Index, *DAR* Delta:Alpha Ratio, *DTR* Delta:Theta Ratio, *DTAR* Delta:Theta:Alpha Ratio, *DTABR* Delta:Theta:Alpha:Beta Ratio, *PRI* Power Ratio Index, *BSI* Brain Symmetry Index, *BBSI* Bilateral Brain Symmetry Index, *r-BSI* Revised Brain Symmetry Index, *pdBSI* Pairwise derived Brain Symmetry Index, *FFT* Fast Fourier Transform, *DWT* Discrete Wavelet Transform, *AUC* Area Under the receiving operator characteristics Curve, *EOG* Electrooculogram, *MRI* Magnetic Resonance Imaging, *DWI* Diffusion Weighted Imaging, *CT* Computed Tomography, *LMCA* Left Middle Cerebral Artery, *MCA* Middle Cerebral Artery, *SAH* Subarachnoid Haemorrhage

^a^novel reanalysis of data from Finnigan 2016 [33]

^b^No further details were reported

Fifteen articles examined differences between stroke from healthy controls, or an identified healthy control dataset, and two compared stroke with stroke mimic conditions [22, 23]. Median article quality score was 3 (range 2–5), but even higher quality reports included only modest numbers of patients (e.g. ischaemic stroke cases ranged from 6–65 patients).

Twelve articles used absolute or relative power ratio computation as a qEEG biomarker. Greater delta power alone could differentiate between stroke and control in 11 articles [12, 21, 25, 27, 29–33, 36, 37]. Less alpha power was associated with stroke in six articles [21, 23, 29, 30, 32, 33]. Less beta activity was associated with stroke versus non stroke in three articles [21, 29, 33]. In one article [23], stroke was associated with less high-beta (20.5-28 Hz) but greater low-beta frequencies (12.5-16 Hz). Increased theta power could identify stroke in three articles [32, 33, 37] but this was in the reverse direction for two others [21, 25].

Stroke was associated with a greater Delta:Alpha Ratio (DAR) in six articles [25, 29–31, 33, 35] greater Delta:Theta Ratio (DTR) in one article [25], and greater Delta:Theta:Alpha:Beta Ratio (DTABR) in three articles [30, 31, 33] but two articles reported that none of these EEG indices were useful [22, 26]. One article [29] showed that stroke was associated with greater Power Ratio Index (PRI) than control patients, indicating a relative increase in ‘slow’ (delta + theta) activity compared with ‘fast’ (alpha + beta) activity. Five articles used indices of brainwave symmetry between hemispheres, such as the Brain Symmetry Index (BSI) [26, 28, 30, 31, 34]. In all but one [30], greater asymmetry was shown for stroke vs control.

Amongst eight articles that calculated any summary indicator of diagnostic accuracy, performance was generally good or high [21, 23, 28, 31, 33, 35–37]. Two articles in particular displayed very high accuracy for individual EEG frequency bands but were not in complete agreement [21, 33]. Within 24 h of symptom onset, Finnigan (2016) [33] reported ischaemic stroke (*n* = 18) could be detected by greater delta (AUC 0.99) and theta (AUC 0.81) activity, but less alpha (AUC 0.97) and beta (AUC 0.90) compared with control patients (*n* = 28). However, although Rogers (2019) [21] also reported accurate prediction by greater delta activity (AUC 0.87) within 72 h onset, there was no difference between stroke (*n* = 10) and controls (*n* = 10) for alpha and beta, and controls had greater theta activity (AUC 0.93). Finnigan (2016) [33] also reported very high AUC from higher DAR (AUC 1.0) and DTABR (AUC 0.99). Subsequent analysis confirmed that the DAR result could be replicated by using just two frontal electrodes (AUC 0.99) [35]. A more recent article used deep learning network-based modelling of clinical information and EEG data from electrode pairs selected by lasso regression within 24 h of symptom onset, and showed the AUC was higher (0.88) than could be achieved by standard analysis of clinical and/or EEG data [23], with slower frequencies in stroke (*n* = 50) and TIA patients (*n* = 13) versus control (*n* = 37).

For two articles also aiming to distinguish stroke from TIA, median quality score was 4 (range 3–5), with small numbers of participants. One article [21] distinguished between stroke and TIA (as well as control) with high diagnostic accuracy using evoked potentials and spectral power across all bands, with greater delta, less alpha and less beta in stroke versus TIA. The other [26] distinguished stroke from TIA using a modified BSI but did not find any difference in slow:fast wave ratios.

#### 1b) Identification of ischaemic versus haemorrhagic stroke

Only two studies considered differences between ischaemic and haemorrhagic stroke aetiologies, with differing methodologies and results. Studies are summarised in Table 2, grouped by year of publication and reference standard.

**Table 2:** EEG during acute clinical assessment to identify ischaemic versus haemorrhagic stroke

**Reference**	**Reference standard**	Participants	Key exclusions	First EEG start time after onset (h)	EEG procedure	EEG processing	EEG biomarker	Result	Quality score
**Vespa 2003 **[38]	**CT/Specialist opinion**	46 Ischaemic Stroke patients, 63 Haemorrhagic Stroke patients	Traumatic head/brain injury, SAH	< 24	Bedside	Online (hospital staff) or offline (EEG segment review or total power trend seizure detection method) seizure detection and classification (focal, hemispheric or generalised)	Epileptiform activity	Haemorrhagic patients exhibited more electrographic seizures (27.8%) vs ischaemic patients (6%) (OR 5.7, 95% CI 1.4 to 26.5, *p* < 0.004; AUC = 0.61, sensitivity = 28.6%, specificity = 93.5% for haemorrhage) (diagnostic accuracy extrapolated from true and false positive and negative values)	4
**Rodriguez 2012 **[29]	**CT/Specialist opinion**	29 Ischaemic Stroke patients, 15 Haemorrhagic Stroke patients (all MCA)	Not Reported	< 72	Not Reported	Not Reported	Relative spectral power (all bands, DAR, power ratio index)	Ischaemic and haemorrhagic stroke significantly differed in the alpha–beta range (earlier, more abrupt decrease in haemorrhage) but PRI and DAR did not differentiate between stroke subtypes Versus normative data, Haemorrhagic patients exhibited a significant decrease in frequencies > 8.59 Hz and Ischaemic patients exhibited significant increase in frequencies < 6.25 Hz and significant decrease in alpha–beta (> 9.38 Hz). (Abstract only—no statistics given)	3

*DAR* Delta:Alpha Ratio, *PRI* Power Ratio Index, *OR* Odds Ratio, *CI* Confidence Interval, *CT* Computed Tomography, *SAH* Subarachnoid Haemorrhage, *MCA* Middle Cerebral Artery

Both studies were of medium quality (median score 3.5, range 3–4). One was an examination of post-stroke seizures during EEG monitoring and found a higher incidence of these was predictive of haemorrhagic stroke [38]; extrapolated specificity was high but sensitivity low. The other used relative spectral power methods and found differences in global frequencies (i.e. a more abrupt decrease of higher frequencies in haemorrhage), but did not find any useful diagnostic value in ratios such as PRI or DAR [29].

#### 1c) Identification of ischaemic stroke due to anterior large vessel occlusion

Five studies reported whether EEG data was associated with direct (angiographic; *n* = 1) or indirect (infarct volume; *n* = 4) radiological evidence that LVO was likely to be responsible for ischaemic stroke. Studies are summarised in Table 3, grouped by year of publication and reference standard.

**Table 3:** EEG during acute clinical assessment to identify radiological changes associated with large vessel occlusion (LVO)

**Reference**	**Reference standard**	Participants	Key exclusions	First EEG start time after onset (h)	EEG procedure	EEG processing	EEG biomarker	Result	Quality score
**Wang 2013 **[39]	**CT and/or MRI**	110 Ischaemic Stroke patients (various lesion sizes)	Cognitive impairment; psychiatric disorders; traumatic brain injury; tumour; encephalitis; hydrocephalus; autoimmune disorders; brainstem stroke	< 10	16 electrodes	Sampling 250 Hz, offline filter 0.5-50 Hz, computer, visual and EOG artifact removal, 2 s epochs,	Relative spectral power (beta only)	Larger infarct size associated with lower beta power (r_1_ = − 0.88881, *P* < 0.001)	3
**Shreve 2019 **[22]	**CT and/or MRI**	6 small infarct Ischaemic Stroke patients, 3 TIA patients, 5 large supratentorial infarct Ischaemic Stroke patients, 10 Stroke Mimic patients	Haemorrhagic stroke	< 43.5	256 electrodes but 62 excluded, awake, fixed gaze with bed at 30 degree angle	Offline only sixth order < 50 Hz filter, independent component analysis artifact removal, 1 s epochs	Relative spectral power (All bands, global power, DAR, DTABR)	Compared to all other groups, large infarcts were associated with higher delta (*p’s* = 0.004–0.038) and DAR in both hemispheres (*p’s* = 0.0006–0.005), greater DTABR (*p* = 0.015) and lower beta (*p* = 0.04) in the contralesional hemisphere	4
**Finnigan 2004 **[12]	**Only MRI (DWI) 15 h ± 3 h**	11 Ischaemic Stroke patients (MCA; PCA; ICA)	Fever, encephalitis, seizures, ICH, non-cortical stroke, confounding neurological condition (e.g. previous stroke) or medication	< 9	64 electrodes, between MRI scans	Online filter .01-100 Hz, artifacts 0.2- 40 Hz, automatic artifact removal, 4 s epochs, sampling 500 Hz, FFT .5-50 Hz	Relative spectral power (aDCI)	Larger infarct size associated with higher aDCI (rho = 0.62, *P* < 0.05)	3
**Wolf 2016 **[40]	**Only MRI**	69 Ischaemic Stroke patients	Seizure	< 48	10–20 system	EEG abnormalities identified	Epileptiform activity (generalised or focal slowing or epileptiform potentials)	Abnormal EEG (*p* = 0.002) and focal EEG slowing (*p* = 0.013) associated with larger territorial infarcts (versus lacunar and embolic)	2
**Erani 2020 **[23]	**Angiography (unclear modality)**	43 Ischaemic Stroke patients (7 LVO), 7 Haemorrhagic Stroke patients, 13 TIA patients	Not Reported	< 23	17 electrodes, portable, dry electrode system, eyes open, resting	Offline analysis (filtering and artifact removal) and re-referencing for bipolar montage	Relative spectral power (all bands, beta split into low and high)	Deep learning EEG (2 lasso selected electrode pairs) and clinical data model (AUC = 0.86, sensitivity = 76%, specificity = 80%) could identify stroke with LVO more accurately than combined clinical and EEG (2 electrode pairs) data (AUC = 0.78, sensitivity = 57%, specificity = 80%), and individual EEG (2 electrode pairs) (AUC = 0.69, sensitivity = 41%, specificity = .80%) or clinical (AUC = 0.80, sensitivity = 65%, specificity = 80%) data models.compared with all other stroke/TIA patients. Greater low frequencies (theta) and lower high frequencies (alpha) associated with LVO	4

*DAR* Delta:Alpha Ratio, *DTABR* Delta:Theta:Alpha:Beta Ratio, *aDCI* acute Delta Change Index, *NIHSS* National Institute of Health Stroke Scale, *CT* Computed Tomography, *MRI* Magnetic Resonance Imaging, *DWI* Diffusion Weighted Imaging, *MCA* Middle Cerebral Artery, *PCA* Posterior Cerebral Artery, *ICA* Internal Carotid Artery; TIA: Transient Ischaemic Attack, *ICH* Intracerebral Haemorrhage

The quality of these studies was mixed, with a median score of 3 (range 2–4). Four reported that relative spectral power detected large infarct volume (more common in LVO), either by identifying areas of increased slower-waves (delta [12, 22] and theta [23]) and/or decreased fast-waves (beta [22, 39] and alpha [23]). Epileptiform activity (including slowing of frequencies) differentiated between territorial infarcts more typical of LVO and sub-cortical infarcts more likely to result from small vessel ischaemia [40]. Two studies comparing activity between hemispheres showed a general trend towards increased slow waves in the affected hemisphere but also a reduction in faster waves in the contralesional hemisphere when infarct size was greater [22, 40]. The only study with direct angiographic evidence of LVO [23] used deep learning models combining clinical and EEG data, showing that the combination could achieve a high level of accuracy to detect 7 cases of LVO amongst 100 cases of suspected stroke (AUC 0.86, sensitivity = 76%, specificity = 80%).

#### 2) Diagnostic accuracy within 6 h of symptom onset

No diagnostic studies were found which consisted purely of patients within six hours of symptom onset. For identification of stroke versus non-stroke, only one small study included patients who were all within nine hours of onset [12], showing significantly greater mean delta power for stroke versus control. Studies which considered ischaemic versus haemorrhagic stroke only included patients within 24 [38] and 72 [29] hours of onset, and no conclusion can be drawn about early EEG application for this purpose. For detection of LVO, two out of five studies involved participants who were potentially within time windows for thrombectomy treatment; within nine [12] and ten [39] hours. These studies did not have high quality scores, but both showed associations with large volume infarction (loss of beta power and higher aDCI respectively) which may indicate that early changes associated with LVO are detectable.

#### 3) Prediction of outcome following confirmed stroke

Twenty-six articles investigated the use of EEG biomarkers in predicting clinical recovery following confirmation of a stroke diagnosis within the previous 72 h. Studies are summarised in Table 4 grouped by year of publication and outcome of interest. Prognostic articles had a median quality score of 4, reflecting a range of scores from poor to excellent (2–5).

**Table 4:** EEG during acute assessment to predict outcomes after confirmed stroke

**Reference**	**Outcome measure**	Participants	Key Exclusions	First EEG start time after onset (h)	EEG Procedure	EEG Processing	EEG Biomarker	Result	Quality score
**Sainio 1983 **[41]	**Admission and 7-day disability**	15 Ischaemic Stroke patients	TIA	< 48	16 electrodes, eyes closed with checking for wakefulness	Online only, > 30 Hz, time constant 0.3 s, sampling 100 Hz	Relative spectral power (all bands), focal and background slowing	Poorer admission outcome associated with background (p = 0.00016) and focal (*p* = 0.0099) abnormalities, greater ipsilesional rolandic and occipital delta_2_ (*p’s* = 0.005) and less ipsilesional rolandic and occipital alpha (*p* = 0.005 and p = 0.025 respectively). Poorer 7-day outcome associated with background abnormalities (*p* = 0.0089) greater ipsilesional (*p *= .025) and contralesional (*p* = 0.025) delta_2_ and less ipsilesional alpha (*p* = 0.025)	4
**Charlin 2000 **[42]	**Day 90 mRS**	47 Ischaemic Stroke Patients	Epilepsy, cirrhosis, cancer, pre-stroke dependence; sedatives	< 24	16 electrodes	None	PLEDs plus and PLEDS proper	Worse outcome (mRS > / = 3) associated with PLEDs (*p* = 0.03, AUC = 0.62, sensitivity = 30.8%, specificity = 93.75%). (prognostic accuracy extrapolated from true and false positive and negative values)	3
**Cuspineda 2003 **[43] ^a^	**mRS at discharge and within three months**	28 Ischaemic Stroke patients (MCA territory)	Haemorrhage	< 72	19 electrodes, awake, eyes open and closed, reclining, temperature controlled	Online filters 0.3-30 Hz, notch 60 Hz, manual artifact removal, 2.56 s epochs	Absolute spectral power (absolute energy)	Discharge and 3-month outcome (mRS) predicted by assessment of EEG absolute energy variables with 100% accuracy (*r* = 0.99). QEEG predicted outcome at discharge better than the CaNS (*p* = 0.03)	2
**Cuspineda 2007 **[44] ^a^	**mRS at discharge and within three months**	28 Ischaemic Stroke patients (MCA territory)	Haemorrhage	< 72	19 electrodes, awake, eyes open and closed, reclining, temperature controlled	Online filters 0.3-30 Hz, notch 60 Hz, manual artifact removal, 2.56 s epochs	Absolute spectral power (all bands Absolute Energy)	Poorer outcome at discharge (mRS) predicted by.less alpha (Accuracy = 92.3% *r* = 0.95) and beta (Accuracy = 69.2%, *r* = 0.76) and greater theta (Accuracy = 92.3%, *r* = 0.94) and delta (Accuracy = 84.6%, *r* = 0.85) power within 24 h. Poorer outcome at 3 months predicted by less alpha (Accuracy = 88.9%, *r* = 0.97) and beta (Accuracy = 77.8%, *r* = 0.83)and greater delta (Accuracy = 88.9%, *r* = 0.92, r 0.87) and theta (Accuracy = 77.8%, *r* = 0.83) within 24-48 h	4
**Sheorajapanday 2011 **[15]	**Day 7 mRS**	60 Ischaemic Stroke patients	Mass lesion; ICH; seizure(s); hypo/hyperglycaemia	Most < 72	19 electrodes, eye closed, awake/alert	Online montage re-referencing; filters > 0.3 Hz, < / = 30 Hz, manual artifact removal, 128 s epochs, FFT	Relative spectral power (DTABR), BSI	Greater DTABR predicted unfavourable outcome (mRS score > = 2) in LACS (AUC = 0.88; accuracy = 0.83%, *p* = 0.01) but not in POCS	5
**Su 2013 **[45]	**Three-month mRS**	162 Ischaemic Stroke patients (large MCA infarct)	Pre-stroke dependence, concurrent illness affecting outcome, sedatives; extraneous factors affecting consciousness	< 72	8 electrodes; pain and auditory stimulation	Online filter 0.5-70 Hz, time constant 0.3 ms	Dominant fast/slow wave with/without reactivity, RAWOD, epileptiform activity, burst and general suppression; alpha/theta coma	Significant associations between worse outcome (mRS > 4) and RAWOD (OR = 2.47, sensitivity = 37%, specificity = 85%) and good outcome and dominant alpha with reactivity (OR = .08, but poor sensitivity = 7.4%, specificity = 49.3%). All other markers had > 80% specificity but < 40% sensitivity in predicting poor outcome. Modified grading most accurate (Kappa = 0.61, *p* = 0.04, sensitivity = 77.9%, specificity = 89.6%, accuracy = 91.4%)	4
**Lima 2017 **[46]	**Three-month mRS**	157 Ischaemic Stroke patients (19 with seizures)	Previous seizures, debilitating neurological disorders, hypo/hyperglycaemia	< 45.5	19 electrodes	None	Epileptiform activity (IED and PP)	Worse outcome (mRS > / = 3) associated with epileptiform activity (OR = 2.94, *p* = 0.001) but not when seizures excluded (OR = 2.13, *p* = 0.07). AUC = 0.60, sensitivity = 51.3%, specificity = 69%). (prognostic accuracy extrapolated from true and false positive and negative values)	4
**Bentes 2017 **[47] ^a^	**mRS (including mortality) at discharge and within 1 year**	151 Ischaemic Stroke patients (ICA; NIHSS 4–42)	Prestroke dependence, traumatic brain injury or surgery, hydrocephalus, history of epilepsy	< 72	64 electrodes, eyes open and closed, resting, hyperventilation and photic stimulation	Not Reported	Asymmetry, Suppression, focal slow-waves, epileptiform activity; periodic discharges	Worse outcome (mRS > / = 3) at discharge associated with EEG background (OR = 5.55, *p* = 0.002) slowing, asymmetry (OR = 11.91, *p* < 0.001) and periodic discharges (OR = 10.39, *p* = 0.027). Worse outcome at 1 year predicted by background slowing (OR = 14.50, *p* < 0.001) and asymmetry (OR = 22.73, *p* > 0.001) and periodic discharges (OR = 14.1, *p* = 0.002). Clinical and radiological predictors plus background asymmetry (AUC = 0.91, sensitivity = 81.1%, specificity = 88.7%) was a better model than clinical data plus past seizures (AUC = 0.83, sensitivity = 72.1%, specificity = 77.5%), clinical (AUC = 0.82, sensitivity = 70.3%, specificity = 73.2%), asymmetry (AUC = 0.81, sensitivity = 72.7%, specificity = 89%) and past seizures (AUC = 0.59, sensitivity = 25.7%, specificity = 93.2%) in isolation. 12-month mortality associated with EEG acute symptomatic seizures (OR = 4.55, *p* = 0.015) and EEG suppression (OR = 7.48, *p* = 0.019). Clinical/radiological predictors plus EEG suppression (AUC = 0.84, sensitivity = 31.8%, specificity = 99.2%) were a better predictor than clinical data plus acute seizures (AUC = 0.82, sensitivity = 40.9%, specificity = 100%), and clinical data (AUC = 0.81, sensitivity = 22.7%, specificity = 98.4%), acute seizures (AUC = 0.64, sensitivity = 0%, specificity = 100%), and suppression (AUC = 0.61, sensitivity = 26.1%, specificity = 96.1%) in isolation	5
**Xin 2017 **[48]	**BI/mRS at 21 days**	29 Ischaemic Stroke patients	TIA, ICH, previous stroke, cardiovascular disorders, traumatic brain injury, tumour, ‘serious disease’, pregnancy	< 72	16 electrodes, < 3 h after meal; sedatives discontinued 3 days prior	Online and offline, filters < 0.53 Hz, > 50 Hz. Sampling 100 Hz, EOG, ECG, EMG, visual and wavelet transform artifact removal, 10 s epochs	r-BSI	Worse outcome (lower BI and higher mRS) associated with higher r-BSI at admission (BI -2.070, *P* = 0.049, mRS 2.256, *P* = 0.033)	3
**Bentes 2018 **[49] ^a^	**mRS at discharge and one year**	151 Ischaemic Stroke patients (ICA;NIHSS 4–42)	Prestroke dependence, traumatic brain injury or surgery, hydrocephalus, history of epilepsy	< 72	64 electrodes, eyes open and closed, resting, hyperventilation and photic stimulation	Offline filters < / = 0.5 Hz, > 70 Hz, notch 50 Hz, manual and automatic artifact removal, 2.05 s epochs; FFT	Absolute spectral power (all bands, DAR, DTABR); BSI	Worse outcome (mRS > / = 3) associated with greater delta (discharge AUC = 0.812, OR = 125; 12 months AUC = 0.836, OR = 129.8), and DTABR (discharge AUC = 0.827, OR = 1.702; 12 months AUC = 0.859, OR = 1.668) and less alpha (discharge AUC = 0.814, OR = 0.221; 12 months AUC = 0.852, OR = 0.16) and beta (discharge AUC = 0.803, OR = 0.28; 12 months AUC = 0.829, OR = 0.28) power (all *p *> 0.001; theta not significant). The best discharge models combined clinical/radiological predictors with background asymmetry (AUC = 0.831, sensitivity = 81.3%, specificity = 68%), DTABR (AUC = 0.827, sensitivity = 87.5%, specificity = 60%), alpha power (AUC = 0.756, sensitivity = 86.9%, specificity = 46.2%) and background slowing (AUC = 0.787, sensitivity = 82.3%, specificity = 60%). The best 12-month models combined clinical/radiological predictors with background asymmetry (AUC = 0.89, sensitivity = 81.1%, specificity = 88.7%), background slowing (AUC = 0.866, sensitivity = 78.4%, specificity = 87.3%), DTABR (AUC = 0.859, sensitivity = 79.7%, specificity = 74.6%) and alpha (AUC = 0.852, sensitivity = 75.7%, specificity = 78.9%). Isolated clinical data, followed by DTABR and alpha were good predictors (AUC’s = 0.768–0.794, sensitivity = 70.1–76.6%, specificity = 64.4–71.8%) (all *p* > 0.001)	4
**Kuznietsov 2018 **[50]	**21-day mRS**	103 Ischaemic Stroke patients (supratentorial)	Cardiovascular or psychiatric disorders, traumatic brain injury, ICH, tumour, past seizure(s)	< 72	19 electrodes	Offline independent component analysis artifact removal, 60 s epochs; FFT	Absolute and relative spectral power (All bands, RSRP; FORG; IHRA)	Worse outcome post-stroke (mRS) associated with higher RSRP of delta band in contralesional hemisphere > 18.4% (OR = 1.31, *p* = 0.0004; AUC = 0.94, sensitivity = 87.0%, specificity = 87.7%, *p* < 0.0001), lower FORG of alpha band in ipsilesional hemisphere > -0.066 (OR = 29.07, *p* = 0.0224; AUC = 0.74, sensitivity = 67.4%, specificity = 70.0%, *p* < 0.0001) and IHRA of alpha band ≤ -0.066 (OR = 0.01, *p* = 0.0402; AUC = 0.66, sensitivity = 60.9%, specificity = 70.2%, *p* < 0.0039). No significant differences for other biomarkers	3
**Rogers 2020 **[51]	**30 and 90-Day mRS and mBI**	12 Ischaemic Stroke patients, 4 Haemorrhagic Stroke patients	Neurological/psychiatric disorders	< 72	Single electrode at 10–20 FP1, eyes closed	Online sampling and amplification, Offline filter 0.5-30 Hz, manual and automatic artifact removal, 4 s epochs; FFT	Absolute and relative spectral power (all bands, DAR, DTR, DTABR)	Only relative theta power significantly negatively correlated with mRS (30-day *r* = -0.54; 90-day *r* = -0.53) and positively with mBI (30-day *r* = 0.60; 90-day *r* = 0.45). Better outcome post-stroke (mBI > / = 95; mRS < / = 1) associated with higher theta values > = 0.25 for 30-day mRS (AUC = 0.81, sensitivity = 71.4%, specificity = 88.9%, *p* = 0.04), mBI (AUC = 0.90, sensitivity = 83.3%, specificity = 90%, *p* < 0.01) and 90-day mBI (AUC = 0.82, sensitivity = 80%, specificity = 81.8%, *p* = 0.05) but not 90-day mRS (AUC = 0.75, sensitivity = 62.5%, specificity = 87.5%, *p* = 0.09). EEG theta power was a no more accurate predictor than NIHSS	4
**Juhasz 1997 **[52]	**Modified NIHSS at 1 month**	40 Ischaemic Stroke patients	Bilateral stroke	< 48	16 electrodes	Online filters < / = 0.3, > 30, 4 s and 80 s epochs, artifacts removed	Absolute spectral power (alpha, beta); APF	Worse outcome (NIHSS) post stroke significantly associated with > 0.5 Hz difference in interhemispheric APF (*p* < 0.02)	3
**Vespa 2003 **[38]	** < 72 h NIHSS and GOS at discharge**	46 Ischaemic Stroke patients, 63 Haemorrhagic Stroke patients (NIHSS 8–42)	Traumatic haemorrhage, SAH, ICH; Brainstem stroke	< 24	14 electrodes	Online (hospital staff) or offline (EEG segment review or total power trend) seizure detection and classification (focal, hemispheric or generalised)	Epileptiform activity	EEG seizures showed no association with GOS 4–5 (*p* = 0.25) but differed significantly according to NIHSS < 72 h (*p* = 0.05)	4
**Finnigan 2004 **[12]	**30 Day NIHSS**	11 Ischaemic stroke patients	Fever, encephalitis, seizures, ICH, non-cortical stroke, confounding neurological condition (e.g. previous stroke) or medication	< 9	64(62) electrodes, alert or drowsy	Online filter .01-100 Hz, artifacts 0.2- 40 Hz, automatic artifact removal, 4 s epochs, sampling 500 Hz, FFT .5-50 Hz	Relative spectral power (aDCI)	Worse outcome (higher NIHSS) associated with greater aDCI (rho = 0.80, *P* < 0.01)	3
**Finnigan 2007 **[53]	**30 Day NIHSS**	13 Ischaemic Stroke patients	Fever, encephalitis, seizures, ICH, confounding neurological condition (e.g. previous stroke) or medication	< 52	62 electrodes, alert or drowsy	Online filter .01-100 Hz, artifacts 0.2- 40 Hz, EOG artifact removal, 4 s epochs, sampling 500 Hz, FFT .5-50 Hz	Relative spectral power (delta, theta, alpha; beta); DAR	Worse outcome (NIHSS) was associated with greater DAR (*r* = 0.91, *P* < 0.001) and less relative alpha power (*r* = -0.82, *P* < 0.01). These correlations were also observed in a 19-channel subset	3
**Wolf 2016 **[40]	**Admission and discharge NIHSS**	69 Ischaemic Stroke patients	Epileptic seizures	< 48	10–20 system	Not Reported	Epileptiform activity; focal slowing	Worse outcome post-stroke (deterioration of NIHSS > 3 points admission vs discharge) associated with generalised EEG slowing (*p* = 0.003)	2
**Yang 2017 **[24]	**7, 14 & 90 Day NIHSS**	86 Ischaemic Stroke patients (NIHSS 4–24)	Cardiovascular disorders, pregnancy	< 4.5	20 electrodes	Online filter .16-70 Hz, sampling 250 Hz, FFT	Relative spectral power (DAR, DTABR), BSI	Neurological improvement of patients post-thrombolysis (decrease in NIHSS by 8 points or return to normal) significantly associated with early decrease in BSI (2 h), DAR (2 h) and DTABR (24 h) (both *p* < 0.01)	4
**De Herdt 2018 **[54]	**Day 7 NIHSS**	29 Ischaemic Stroke patients, 2 Haemorrhagic stroke patients	Not Reported	< 72	Not Reported	Not Reported	Epileptiform activity (spikes, spike-waves; seizure, PLEDs)	Epileptiform activity not associated with outcome, only useful for predicting seizure incidence (abstract only—no statistics provided)	2
**Gur 1994 **[55]	**Dementia diagnosis, checked every 6 months for 2 years**	199 Ischaemic Stroke patients	Cognitive impairment, TIA, ICH, previous stroke	< 48	18 electrodes	Not Reported	Abnormal EEG patterns, foci, background slowing	Worse outcome (development of dementia) associated with abnormal EEG (OR = 2.6, *p* = 0.003, AUC = 0.38, sensitivity = 63.4%, specificity = 12.2%) (prognostic accuracy extrapolated from true and false positive and negative values)	3
**Wang 2013 **[39]	**MoCA at two weeks and 2 years**	110 Ischaemic Stroke patients	Cognitive impairment, psychiatric disorders, traumatic brain injury, tumour, infection, multi-infarct, systemic disease, psychoactive drug use	< 10	16 electrodes	Sampling 250 Hz, offline filter 0.5-50 Hz, computer, visual and EOG artifact removal, 2 s epochs,	Relative spectral power (beta only)	Significantly lower beta power with cognitive impairment and larger infarct size (P < 0.01). Sensitivity: 92.3% for predicting impairment and 93.3% for predicting normal cognition. Good concordance between MoCA scores and beta power (Kappa statistic = 0.851, *p* < 0.001)	3
**Song 2015 **[56]	**MoCA (Beijing version) 11 months—7 years**	105 Ischaemic Stroke Patients	Cognitive impairment, psychiatric disorders, traumatic brain injury, tumour, infection, multi-infarct, systemic disease, psychoactive drug use	< 12	16 electrodes, eyes closed with checking for wakefulness	Online filter 0.5-50 Hz, Offline 2 s epochs, EOG artifact removal, FFT	Relative spectral power (all bands)	Worse outcome associated with high background rhythm frequency (HR = 14 (3.8, 41), *P* < 0.001) or greater median theta power (HR = 5 (1.4, 7.8), *P* = 0.002)	4
**Aminov 2017 **[25]	**90 Day MoCA**	15 Ischaemic Stroke patients, 4 Haemorrhagic Stroke patients	Neurological/psychiatric disorders, previous stroke	< 72	Single electrode at FP1, eyes closed	Online filter 0.5-30 Hz, manual artifact removal, 4 s epochs; FFT	Relative spectral power (DAR, DTR)	Better outcome moderately correlated with higher relative theta power (r = 0.50, p = 0.01), lower DAR (*r* = -0.45, *p* = 0.03), DTR (*r* = -0.57, *p* = 0.01) and relative delta power (*r* = -0.47, *p* = 0.02)	4
**Yan 2011 **[28]	**Mortality**	22 Stroke patients	Not Reported	< 48	16 electrodes, eyes closed, resting	Offline visual artifact removal followed by digital filter, 10 s epochs. FFT	BBSI	BBSI > 0.082 predicted mortality with an accuracy of 86.36%	2
**Chen 2018 **[30]	**Mortality at Day 90**	47 Haemorrhagic Stroke patients	Aneurysm, vascular malformation, traumatic head/brain injury, tumour, infection/encephalitis	< 59	16 electrodes, eyes closed and awake; supine	Offline filters > 0.3, < / = 30 Hz, artifacts removed. FFT	Relative spectral power delta, alpha, DAR, DTABR), BSI	Mortality at Day 90 was associated with higher DAR (OR 5.306, *p* = 0.008). AUC for TCD-QEEG(DAR) model = 0.949	4
**Jiang 2019 **[57]	**Mortality at discharge and six months**	58 Ischaemic Stroke patients	Prestroke dependence, consciousness altering drugs, haemorrhage, tumour, encephalitis, epilepsy	< 72	16 electrodes	Online filters 0.5-30 Hz and offline visual artifact rejection. FFT	Relative spectral power (All bands, DTABR), BSI	Mortality at discharge and six months post-stroke associated with greater contralateral electrode theta power > / = 25.53 (discharge *p* = .038, accuracy = 68%, sensitivity = 69.2%, specificity = 66.7%), 6-month *p* = 0.026, accuracy = 64%, sensitivity = 45.2%, specificity = 94.7%). No other biomarkers significantly contributed to the model	4

*DAR* Delta:Alpha Ratio, *DTR* Delta:Theta Ratio, *DTABR* Delta:Theta:Alpha:Beta Ratio, *APF* Alpha Peak Frequency, *Adci* Acute Delta Change Index, *RSRP* Relative Spectral Rhythm Power, *BSI* Brain Symmetry Index, *BBSI* Bilateral Brain Symmetry Index, *r-BSI* Revised Brain Symmetry Index, *IHRA* Interhemispheric Rhythm Asymmetry, *FFT* Fast Fourier Transform, *RAWOD* Regional Attenuation Without Delta, *FORG* Front-Occipital Rhythm Gradient, *PLEDs* Periodic Lateral Epileptiform Discharges, *IED* Interictal Epileptiform Discharge, *PP* Periodic Patterns, *OR* Odds Ratio, *HR* Hazard Ratio, *AUC* Area Under the receiving operator characteristics Curve, *mRS* Modified Rankin Score, *BI* Barthel Index, *mBI* Modified Barthel Index, *CaNS* Canadian Neurological Scale, *GOS* Glasgow Outcome Scale, *MoCA* Montreal Cognitive Assessment, *NIHSS* National Institute of Health Stroke Scale, *TCD* Transcranial Doppler, *QEEG* Quantitative EEG, *EMG* Electromyogram, *EOG* Electrooculogram, *ECG* Electrocardiogram, *LACS* Lacunar Stroke, *POCS* Posterior Circulation Stroke, *ICH* Intracerebral Haemorrhage, *SAH* Subarachnoid Haemorrhage, *TIA* Transient Ischaemic Attack, *ICA* Internal Carotid Artery, *MCA* Middle Cerebral Artery

^a ^Two pairs of papers (Bentes 2017 and 2018 [47, 49]; Cuspineda 2003 and 2007 [43, 44]), appear to be separately reporting different data from the same overall cohorts of 151 and 28 patients respectively

Twelve articles assessed outcome at various time points by dependency scales such as the modified Rankin Scale (mRS), Barthel Index/modified Barthel Index (BI/mBI), Glasgow Outcome Scale (GOS) or disability via “neurological examination”. Of these, six found associations between post-stroke dependency and generalised abnormal EEG patterns such as asymmetry, slowing and epileptiform activity [41, 42, 45–47, 49]. One study found no association between EEG-recorded seizures and GOS [38]. Another eight found associations between spectral power (alpha, theta and delta) and/or spectral power ratios and poor outcome, including: more delta activity [41, 44, 49, 50], less alpha activity [41, 44, 49, 50], greater theta activity [41, 44, 50, 51] but also less theta activity [49], greater DTABR [15, 49] and higher Brain Symmetry Index score (denoting greater asymmetry) [48]. One study stated that qEEG frequencies predicted outcome but did not provide further detail [43]. Prognostic accuracy was variable amongst 10 articles with accuracy indices [15, 42–47, 50, 51, 58], and boosted by inclusion of clinical and radiological predictors [47, 49].

Seven articles assessed later neurological impairment using the NIHSS. Of three studies seeking associations with abnormal EEG patterns, one found an association with epileptiform activity [38] and one generalised EEG slowing [40]. One study [54] did not report an association between epileptiform activity and the NIHSS, finding this was only useful in predicting seizure incidence. Three studies found associations between poor outcome and relative band power or ratios using various biomarkers such as less relative alpha power and greater DAR [53], greater interhemispheric alpha peak frequency asymmetry [52] and greater aDCI [12]. One study showed associations between a more favourable NIHSS after thrombolysis for ischaemic stroke and early decreases in BSI, DAR and DTABR [24]; this was the only study to focus solely on patients within six hours of symptoms onset. None of these studies calculated summary statistics to reflect accuracy.

Four studies assessed outcome by cognitive function (MoCA or diagnosis of dementia). Three found associations between spectral power and poorer cognitive outcome: lower theta, higher delta, greater DTR and DAR [25], lower beta [39], and greater theta with high background rhythm frequency [56]. One older study simply associated “abnormal EEG”, such as abnormal foci and background slowing, with risk of developing dementia [55]. Prognostic accuracy was low for any ‘abnormal EEG’ recording [55] but high (sensitivity 92.3%) if low beta activity was present [39].

Four studies considered prediction of mortality at various time intervals after stroke. At hospital discharge, greater contralateral theta power [57] and greater asymmetry measured by the Bilateral Brain Symmetry Index (BBSI) [28] were associated with poorer outcome. Higher DAR at day 90 [30], greater contralateral theta power at 6 months [57], and epileptiform activity, background slowing and overall asymmetry at 12 months [47] were associated with poorer outcome. Prognostic accuracy was moderate, but with poor sensitivity, in two studies [47, 57] and appeared high for two other studies [28, 30].

## Discussion

In this review, we have summarised published literature on the use of EEG in the diagnosis and prognosis of stroke when applied within 72 h of onset. Due to variability in study design and EEG technology, we did not plan to directly compare clinical utility of EEG biomarkers or perform a meta-analysis. Despite limitations in study quality, such as unclear inclusion criteria and reference standards, reports generally support further development and evaluation of EEG techniques to examine their ability to facilitate accurate clinical stratification of patients with stroke symptoms.

There is evidence to support potentially valuable diagnostic accuracy of EEG approaches for differentiating stroke from non-stroke states due to statistical associations between a diagnosis of stroke, increased slow-wave EEG activity (delta in particular) and decreased fast-wave activity (alpha and beta). Although theta activity was often increased for stroke relative to control subjects, this was not a consistent finding and it appears to be the least useful frequency for diagnosis in this context, probably due to its intermediate speed between alpha (fast) and delta (slow). Two studies found greater asymmetry and slow-wave activity in stroke versus TIA, but it should be noted that both recruited patients within 72 h of symptom onset, during which TIA symptoms would have resolved, and so the results would have little value in discriminating stroke versus TIA or in assisting with therapeutic decision making. However, EEG may have value in differentiating TIA and stroke mimic patients which could have implications for future secondary prevention. Despite these promising early studies, it is important to recognise that most were small and included selected patients who were beyond six hours since symptom onset, so there is relatively little evidence that the potential EEG biomarkers identified would be present in the very early stages of stroke when the impact for emergency care decisions would be greatest e.g. to initiate direct ambulance transfer to a stroke centre rather than a general hospital without specialist care.

Evaluation of the ability of EEG to distinguish between haemorrhagic and ischaemic stroke was limited to two studies, which again were not focussed upon the early hours when this information would be of greatest clinical value e.g. for administration of thrombolytic therapy within 4.5 h of symptom onset [38]. Because stroke due to haemorrhage typically exhibits greater symptom severity at presentation [59] and as neither study adjusted for patient characteristics, it is also unknown whether the observed EEG differences are attributable to the underlying stroke type. Currently it appears unlikely that EEG has a role to play in the differentiation between ischaemic and haemorrhagic stroke that would change patient management.

On the basis that indirect radiological evidence is a reliable indicator of LVO, a small number of studies support the further development of EEG biomarkers for this purpose, although only two studies focused on a suspected stroke population during the standard time interval of maximal clinical value for thrombectomy [12, 39]. For all studies, it was unclear whether participants were fully representative of an unselected suspected stroke population, and it remains uncertain whether it will be possible to accurately transfer suitable patients directly to thrombectomy centres. Only one study used angiography as the reference standard and reported that the most promising AUC (0.86) was achieved by combining clinical and EEG (lower alpha and greater theta) data with a deep learning algorithm [23]. In addition, a prospective study published since completion of our search has also confirmed that amongst 109 patients within 24 h of symptom onset (25 angiography-proven LVO, 38 non-LVO ischemic, 14 haemorrhages, and 32 stroke mimics) an AUC of 0.88 was achieved using a portable LVO-detection device which combined EEG and somatosensory-evoked potentials [60].

When used to provide an early estimate of prognosis, EEG biomarkers recorded within 72 h of stroke onset had associations with later clinical outcomes which could be useful to inform acute management decisions including future dependency, neurological impairment, cognitive function and mortality. In particular, greater delta and theta activity, less alpha and beta activity, greater interhemispheric asymmetry and greater DAR and DTABR ratios appear to be moderate predictors for both long-, and short-term neurological function and dependency. Such associations are not unexpected as EEG changes reflect the volume of cerebral tissue injury, which itself directly correlates with dependency and survival [61]. Only one cohort was identified where EEG information improved upon the accuracy of predictions for dependency and mortality made using simple clinical assessments and/or brain imaging to confirm the number and location of vascular lesions [47, 49]. There are, however, validated clinical scores already available to estimate various aspects of physical stroke recovery (e.g. arm function [62]; independent walking ability [63]) which are not widely used in practice because of concerns that they could restrict access to finite, but potentially beneficial, care resources [64]. Therefore, in parallel with further research focussed upon whether surface EEG can refine early clinical prediction of future survival and dependency, it is necessary to understand whether using such technology as a decision support aid is an acceptable concept and how the results would be communicated to patients and their families. Likewise, although clinical models to predict cognitive impairment after stroke have been created, currently there is insufficient evidence of validity and/or accuracy for routine use [65]. Neuroimaging variables such as white matter lesions have separately been found to be risk factors for dementia after stroke [66] and so it is feasible that EEG biomarkers will be helpful in identifying patients with a subclinical risk. However, studies identified by our search did not combine EEG data with neuroimaging variables, or compare to age matched controls, and it will be necessary to undertake additional longitudinal studies of well described cohorts before it is clearer whether EEG has a role to play as a clinical decision aid by providing prognostic estimates during acute stroke care.

According to the basic scoring system we employed, most studies were not high quality, usually due to a lack of clarity about populations, reference standards and adjudication. Few studies produced power calculations or seemed to be adequately powered given required case:variable ratios. Many studies did not calculate prognostic or diagnostic accuracy or provide information that would be important to determine clinical utility, such as the number of patients who could not tolerate the procedure and the time required to obtain an EEG recording. Techniques using large numbers of electrodes are unlikely to be deployed during emergency assessment of suspected stroke if application requires additional training and significantly delays routine care, but it is encouraging that diagnostic value was reported by studies using six electrodes or fewer [21, 34, 35]. Clinical feasibility will be further facilitated by easily applicable dry (sans electroconductive gel) electrode systems, and ongoing development of machine learning approaches to automatically select electrode pairs and rapidly identify multi-wave activity patterns predictive of a stroke diagnosis or LVO [23, 60]. Rapid application is less essential for collecting information to inform prognosis and could be done after hospital arrival, but it is still important to consider that some patients may not be able to tolerate a lengthy EEG procedure and efficient portable systems will minimise disruption of acute clinical care.

As well as high accuracy and rapid electrode application, other characteristics have been described that would make diagnostic technology more suitable for prehospital use including: a user-friendly interface with an easily interpretable output that enables operation by ambulance practitioners with relatively low exposure to stroke, a rugged compact design enabling storage in ambulances, and a cost which is affordable relative to the impact upon patient care and outcomes [67]. For prehospital EEG deployment, it would also be important that ambulance practitioners receive clear instruction about which suspected stroke patients should have a reading taken. For instance, Bayesian modelling has shown that because ischaemic stroke patients with low NIHSS scores are unlikely to have LVO whilst those with high scores are very likely to have LVO, a diagnostic would have greatest population level impact when used for patients with intermediate symptom severity e.g. (Additional file 1) NIHSS scores of 5 to 10 points [68]. Other considerations influencing the utility of diagnostic technology during prehospital assessment are how often patients develop acute complications (including seizures), the prevalence of pre-existing co-morbidities that limit emergency treatment options, and the location of the incident relative to the nearest stroke centre.

### Limitations

Finally, our review has some limitations which should be acknowledged. It was not possible to include studies written in a non-English language, which may have excluded relevant reports that did not already have an English translation available. There was a wide variation in EEG technique (e.g. filters and electrode placement) and outcome measures which prevented data meta-analysis and hinders recommendation of a specific technical approach for diagnosis or prognosis. Additionally, these cohorts were specifically selected for research and most studies had strict inclusion criteria to minimise interference with the EEG signal, limiting the generalisability of findings to the wider suspected stroke population, especially in the prehospital setting. In many studies it was unclear which diagnostic reference standards were used and even when these were described, a lack of standardisation limited interpretation of the results. Lastly, as none of these studies were conducted in a prehospital setting, the findings cannot be directly extrapolated to the more challenging ambulance environment, where useful diagnostic technology should be able to detect very early changes of brain tissue amongst a heterogeneous population.

### Future directions

There have been recent advances in commercial EEG technology for use in stroke diagnosis, notably for early identification of LVO [18], but our review was limited to published studies. Based on results to date, it is important to continue to evaluate these devices in real world populations to determine which combination of EEG data might assist with early triage of suspected stroke. An ideal diagnostic accuracy study design would prospectively collect blinded EEG readings during prehospital clinical assessment and compare the performance of a predetermined data algorithm to an independently adjudicated reference standard which is appropriate for the intended purpose e.g. in a study to identify LVO, the reference standard would require all ischaemic stroke participants to undergo angiography to confirm or exclude occlusions in pre-specified locations of the cerebral vasculature. The sample size should be justified in advance by a calculation describing a clinically meaningful minimum level of performance, and the participant inclusion criteria should reflect a relevant clinical population e.g. if the purpose is to facilitate thrombolysis, a maximum of 4.5 h should be allowed between symptom onset and EEG reading. Important operational information must be reported to assess feasibility such as technical failures and the time taken to apply electrodes. If satisfactory EEG diagnostic accuracy is demonstrated and the technology appears to be acceptable for clinical deployment, further trials should evaluate the real-world impact upon the efficiency of stroke care pathways, including its overall cost-effectiveness during delivery of emergency therapies. Other portable technologies are also in development for emergency detection of stroke and LVO, including blood assays and non-ionising imaging [8], and the future clinical value of surface EEG should be considered alongside alternative biomarkers used separately and in combination.

## Conclusion

Reports identified during this review show that surface EEG techniques have promise for assisting with stroke diagnosis and prognosis during the acute phase. However due to the small size of studies and variations in technology, populations and settings, it is not yet possible to make recommendations regarding EEG use to guide early diagnostic and prognostic management decisions. Further research is required to determine which combinations of electrodes, waveforms, clinical data and neuroimaging variables can accurately stratify unselected populations into clinically important subgroups, and to confirm that EEG application for this purpose is both acceptable and feasible within the first few hours after symptom onset.

## Supplementary Information

**Additional file 1.**

## Data Availability

Data sharing is not applicable to this article as no datasets were generated or analysed during the current study.

## Abbreviations

aDCI: Acute Delta Change Index
APF: Alpha Peak Frequency
AUC: Area Under the receiving operator characteristics Curve
BI: Barthel Index
BSI: Brain Symmetry Index
BBSI: Bilateral Brain Symmetry Index
CaNS: Canadian Neurological Scale
CI: Confidence Interval
CT/CTA: Computed Tomography/Computed Tomography Angiography
DAR: Delta:Alpha Ratio
DTABR: Delta:Theta:Alpha:Beta Ratio
DTAR: Delta:Theta:Alpha Ratio
DTR: Delta:Theta Ratio
DWT: Discrete Wavelet Transform
DWI: Diffusion Weighted Imaging
ECG: Electrocardiogram
ED: Emergency Department
EEG: Electroencephalogram
EMG: Electromyogram
EOG: Electrooculogram
FFT: Fast Fourier Transform
FORG: Fronto-Occipital Rhythm Gradient
GOS: Glasgow Outcome Scale
HR: Hazard Ratio
ICA: Internal Carotid Artery
ICH: Intracerebral Haemorrhage
IED: Interictal Epileptiform Discharge
IHRA: Interhemispheric Rhythm Asymmetry
LACS: Lacunar Stroke
LMCA: Left Middle Cerebral Artery
LVO: Large Vessel Occlusion
mBI: Modified Barthel Index
MCA: Middle Cerebral Artery
MoCA: Montreal Cognitive Assessment
MRI/A: Magnetic Resonance Imaging/Angiography
mRS: Modified Rankin Scale
NIHSS: National Institutes of Health Stroke Scale
OR: Odds Ratio
PCA: Posterior Cerebral Artery
pdBSI: Pairwise derived Brain Symmetry Index
PLEDs: Periodic Lateral Epileptiform Discharges
POCS: Posterior Circulation Stroke
PP: Periodic Patterns
PRI: Power Ratio Index
PRISMA: Preferred Reporting Systems for Systematic Reviews and Meta-Analyses
RAP: Relative Alpha Power
RAWOD: Regional Attenuation Without Delta
RDP: Relative Delta Power
RDTP: Relative Delta and Theta Power
RSRP: Relative Spectral Rhythm Power
r-BSI: Revised Brain Symmetry Index
SAH: Subarachnoid Haemorrhage
TIA: Transient Ischaemic Attack
TCD: Transcranial Doppler ultrasonography
QEEG: Quantitative EEG
