# Supplementary material for: Surface electroencephalography (EEG) during the acute phase of stroke to assist with diagnosis and prediction of prognosis: a scoping review
Source: BMC Emerg Med. 2022 Feb 28;22:29. doi: 10.1186/s12873-022-00585-w (PMC8883639; doi:10.1186/s12873-022-00585-w)
Supplement: Supplementary file 1 — Additional file 1. [file 12873_2022_585_MOESM1_ESM.docx]

**Supplementary Material**

**Supplement A**

**Search Strategies**

**OVID**

1. Exp Stroke/ OR cerebrovascular accident.mp. OR cva.mp. OR large vessel occlusion OR exp Brain Infarction OR h?emorrhagic stroke.mp.

AND

1. Emergency medical services or exp triage/ OR emergency medical service*.mp. OR paramedic*.mp. OR ems.mp. OR triage.mp. Or triag*.mp. OR pre?hospital*.mp. OR ambulance.mp. OR diagnosis/ or exp clinical decision-making/ or exp delayed diagnosis/ or exp diagnosis, computer-assisted/ or exp diagnosis, differential/ or exp diagnostic errors/ or exp "diagnostic techniques and procedures"/ or exp early diagnosis/ or exp prognosis/ OR diagnos* OR stratification.mp. OR assess*.mp. OR identif*.mp. OR facilit*.mp. OR detect*.mp.

AND

1. EEG.mp. OR qEEG.mp. OR quantitative EEG.mp. OR electroencephalography.mp. OR electro?encephalography.mp. OR continuous EEG monitoring.mp. OR brain symmetry index.mp. OR delta?alpha power ratio.mp. OR delta alpha ratio.mp.

**Web of Science**

Database: ALL DATABASES

1. Stroke OR “cerebrovascular accident” OR CVA OR “large vessel occlusion” OR “brain infarction” OR “h$emorrhagic stroke”

AND

1. “Emergency medical service*” OR paramedic* OR EMS OR triag* OR pre$hospital* OR ambulance* OR “clinical decision-making” OR “delayed diagnosis” OR “computer-assisted diagnosis” OR “differential diagnosis” OR “diagnostic errors” OR "diagnostic techniques and procedures" OR “early diagnosis” OR prognos* OR diagnos* OR stratif* OR assess* OR identif* OR facilit* OR detect*

AND

1. EEG OR qEEG OR “quantitative EEG” OR electro$encephalography OR “continuous EEG monitoring” OR “brain symmetry index” OR “delta$alpha power ratio” OR delta alpha ratio

**Scopus**

Stroke OR “cerebrovascular accident” OR CVA OR “large vessel occlusion” OR “brain infarction” OR “h*emorrhagic stroke”

AND

“Emergency medical service*” OR paramedic* OR EMS OR triag* OR pre*hospital* OR ambulance* OR “clinical decision-making” OR “delayed diagnosis” OR “computer-assisted diagnosis” OR “differential diagnosis” OR “diagnostic errors”

OR

"diagnostic techniques and procedures" OR “early diagnosis” OR prognos* OR diagnos* OR stratif* OR assess* OR identif* OR facilit* OR detect*

AND

EEG OR qEEG OR “quantitative EEG” OR electro*encephalography OR “continuous EEG monitoring” OR “brain symmetry index” OR “delta*alpha power ratio” OR delta alpha ratio

**Supplement B**

**Review extraction tool components**

*1. Study Inclusion Criteria Matrix*

|  |
| --- |
| **1. STUDY DESIGN** |
| **Primary quantitative research studies with abstracts published in English from any country with n >2 patients.** |
| **2. PARTICIPANTS** |
| **Patients with suspected or confirmed stroke, where the EEG intervention was performed within 72hrs.** |
| **3. CONCEPT** |
| **Intervention: EEG-based interventions, where EEG is used in stroke stratification, diagnosis or prognosis, including but not limited to: qualitative visual analysis of EEG, quantitative EEG, continuous EEG monitoring, the Brain Symmetry Index (BSI) and frequency-specific power measures such as delta/alpha power ratio (DAR) or (delta + theta)/(alpha +beta) power ratio (DTABR).** |
| **There must also be either:** |
| **Reference standard - may include other diagnostic methodologies such as MRI/A, CT/A, PET or specialist opinion.** |
| **And / or** |
| **Stroke outcomes: Any validated stroke outcome measure such as NIHSS, mRS, infarct volume.** |
| **For diagnostics: Statistical comparison of stroke patients with a defined reference group (prospective control patients, normative database, mimics or other stroke subtype).** |
| **4. CONTEXT** |
| **Acute hospital care or ambulance, or adjunct locations appropriate to the study, for example patients who were conveyed to a specialist EEG laboratory from hospital for the intervention to be applied.** |
|  |
|  |
| **If Yes for 1 AND 2 AND 3 AND 4 - INCLUDE If No for 1, 2, 3 OR 4 - EXCLUDE (note reason[s] in comments box)**  EEG: Electroencephalography; MRI/A: Magnetic Resonance Imaging/Angiography; CT/A: Computed Tomography/Angiography; PET: Positron Emission Tomography; NIHSS: National Institute of Health Stroke Scale; mRS: modified Rankin Scale. |

*2. Abstract review*

| **Reference no.** | **Do not record name of authors or journal title as these may unconsciously bias reviewers** |  |  |  |  |  |
| --- | --- | --- | --- | --- | --- | --- |
|  | **Title** | **Year** | **Exclude/Review Full Text** | **If exclude, why/which criterion?** | **Discrepancy resolution if required** | **Notes for included** |
|  |  |  |  |  |  |  |
|  |  |  |  |  |  |  |
|  |  |  |  |  |  |  |
|  |  |  |  |  |  |  |
|  |  |  |  |  |  |  |
|  |  |  |  |  |  |  |
|  |  |  |  |  |  |  |
|  |  |  |  |  |  |  |
|  |  |  |  |  |  |  |
|  |  |  |  |  |  |  |
|  |  |  |  |  |  |  |

*3. Search of relevant review articles for additional articles*

| **Review Reference no.** | **Title** | **Year** | **Relevant Abstracts n=** | **Discrepancy resolution if required** | **Notes** |
| --- | --- | --- | --- | --- | --- |
|  |  |  |  |  |  |
|  |  |  |  |  |  |
|  |  |  |  |  |  |
|  |  |  |  |  |  |
|  |  |  |  |  |  |
|  |  |  |  |  |  |
|  |  |  |  |  |  |

*4. Review of full text articles*

| **Ref no.** | **Do not record name of authors or journal title as these may unconsciously bias reviewers** |  | **Criteria assessment** | |  |  | |  |  | | **Comments/**  **Discrepancy resolution if required** | **Decision** |
| --- | --- | --- | --- | --- | --- | --- | --- | --- | --- | --- | --- | --- |
|  | **Title** | **Year** | **1. Study design** | **2. Participants** | **3. Concept - Intervention** | **3. Concept - Reference Standard** | **3. Concept - Outcome** | **3. Concept - Statistics** | **4. Context** | **Include? (Y/N)** |  |  |
|  |  |  |  |  |  |  |  |  |  |  |  |  |
|  |  |  |  |  |  |  |  |  |  |  |  |  |
|  |  |  |  |  |  |  |  |  |  |  |  |  |

*5. Full text review - discrepancy between reviewers*

| Reviewer 1 Included | Reviewer 2 Included | Discussion | Adjudication | Result |
| --- | --- | --- | --- | --- |
|  |  |  |  |  |
|  |  |  |  |  |
|  |  |  |  |  |
|  |  |  |  |  |
|  |  |  |  |  |
|  |  |  |  |  |
|  |  |  |  |  |
|  |  |  |  |  |

*6. Data extraction matrix sections*

| 1. Admin information | |  |  |  |  |
| --- | --- | --- | --- | --- | --- |
| Reference No. | Study title | Date of extraction | Extracted by | Year of publication | Country of origin (Location of lead author) |
|  |  |  |  |  |  |

| 1. Study Methods | | | |  | |  |  | |  | |  | |  | | |
| --- | --- | --- | --- | --- | --- | --- | --- | --- | --- | --- | --- | --- | --- | --- | --- |
| **Study aim** (free text from paper - ie copy and paste if a sentence that appears to be an aim exists. Do not create an aim or cobble together an aim) | | **Study aim categorised** (using all information in the paper including what is reported in results as well as any stated aims categorise as 'diagnostic' (ie EEG being used to make a diagnosis) or 'prognostic' (ie EEG being used to indicate some prognostic category) or 'both'. There should not be unclear here as this is related to the inclusion criteria. However, if one is unclear note this and return later to see if the study was included in error) | | **Study design** (as described in paper eg cross sectional survey, cohort, unclear) | | **Study setting** (ambulance, hospital ED, hospital stroke unit, hospital neurology department, hospital other department, specialist laboratory, other: state, unclear) | **Participant inclusion criteria** (intended criteria ie listed in methods or potentially elsewhere in the paper, free text, if unclear then state this) | | **Participant exclusion criteria** (intended criteria ie listed in methods or potentially elsewhere in the paper, free text, if unclear then state this) | | **EEG technology details** - free text details about the EEG technology used to collect data including how many times it was used eg did some patients get 2 measurements at different time points | | **EEG processing** - free text details about data processing or analysis of EEG after collection, e.g. artefact removal, examination for epileptiform activity, algorithms such as BSI or DTABR | | |
|  | |  | |  | |  |  | |  | |  | |  | | |
|  | | | | | | | | | | | | | | | |
| 1. Study Methods Continued | | | | |  | | |  | |  | |  | |  |  |
| **If study used EEG as a diagnostic, list reference standard test(s)** (CT, CTA, MRI, MRA, other brain imaging, combination of brain imaging, specialist opinion, unclear, not stated) | **If study used EEG as a diagnostic, document timing of reference standard test(s)** (eg 7 days after sympton onset) | | **If study used EEG as a diagnostic and a reference standard test is reported, was this reference standard test clearly defined before the study started** (ie pre-defined before any data was examined) | | **If study used EEG to indicate prognosis, list measurements of prognosis used** (eg NIHSS, Barthel - list all) | | | **If study used EEG to indicate prognosis, document timing of measurements of prognosis used** (eg 7 days after stroke, 3 months after stroke, document for each measurement) | | **If study used EEG to indicate prognosis and measurements of prognosis are reported, were the measures clearly defined before the study started** (ie pre-defined before any data was examined) | | **Blinding - what aspects of the study were blinded** (eg EEG used and result reported without knowledge of diagnosis, reference standard undertaken and reported without knowledge of EEG, prognostic measures undertaken without knowledge of EEG data, etc.) | | **Was a sample calculation undertaken prior to the start of the study?** (yes, no, unclear, not stated) |  |
|  |  | |  | |  | | |  | |  | |  | |  |  |

|  |  |  |  |  |
| --- | --- | --- | --- | --- |
| 1. Study | results |  |  |  |
| **Number of participants enrolled** (i.e. consented or had some study proceedures done - not number screened or number in analyses) | **Time from symptom onset to EEG recording** (i.e. from results data. If more than one EEG list all) | **Results** (list results of **all** analyses undertaken - may be several e.g. sensitivity to detect stroke is x, AUROC curve for x is y) | **For the analyses listed, is the number of participants included in each separate analysis clear?** | **Where the number of participants included in all analyses are clear and the numbers are NOT the number of participants enrolled, is it clear why some people are missing from the analysis** (give reasons for participants excluded if available) |
|  |  |  |  |  |

*7. Quality assessment*

| 1. Were the study eligibility criteria **clearly** defined? |  |  |  |  |  | Yes or No | |  |
| --- | --- | --- | --- | --- | --- | --- | --- | --- |
| 2. Is the EEG technology **clearly** defined? |  |  |  |  |  | Yes or No | |  |
| 3. If the study uses EEG as a diagnostic, was the reference standard(s) **clearly** defined before the start of the study? | | | |  |  | | Yes or no | |
| 4. If the study uses EEG to indicate prognosis, were the prognostic measurement tools (eg NIHSS) **clearly** defined before the start of the study? | | | | | | | Yes or no | |
| 5. Did the study report **any** level of blinding? |  |  |  |  |  | Yes or no | |  |
| 6. Are **all** enrolled participants accounted for in **all** analyses. |  |  |  |  |  | Yes or no | |  |

| **Quality Assessment Table** | | | | | | | | | | |
| --- | --- | --- | --- | --- | --- | --- | --- | --- | --- | --- |
| **Reference no.** | **Title** | **Year** | **Quality criterion (Yes/No)** | | | | | | **Score (0 to 6)** | **Comments** |
|  |  |  | **1. Eligibility** | **2. Technology** | **3. Reference Standard** | **4. Prognostic tool** | **5. Blinding** | **6. Participants accounted for** |  |  |
| Example |  |  | Yes | No | Yes | No | Yes | No | 3 |  |
|  |  |  |  |  |  |  |  |  |  |  |
|  |  |  |  |  |  |  |  |  |  |  |

**Supplement C**

**Table S1: Summary of study inclusion/exclusion criteria**

| CRITERION | Number of studies providing this information | BREAKDOWN  (Number of studies in each category) |
| --- | --- | --- |
| Age | 10 | >18 (7), 18-80 (1), 18-85(1), 35-80(1) |
| Diagnostic stroke status | 24 | Confirmed (14), Suspected (9), Both (1) |
| Stroke subtype | 35 | Ischaemic (29), Haemorrhagic (1), Both (5) |
| Territory/Location | 24 | MCA territory (7), anterior (1), cortical (2), encephalic trunk/brainstem infarct excluded (3), hemispheric (1), ICA territory (2), PCA territory (1), LVO (1), supratentorial (1), unilateral (4) |
| Medical history | 30 | Excluded if: Prior stroke (10), Head/brain injury (1), Tumour/lesion (1), Seizure/epilepsy (4), Combination of these (14), |
| Concomitant medication | 14 | Excluded if: Neurologically active/Affects EEG (14), Thrombolytic/anticoagulant (2) |
| Imaging | 16 | Imaging performed (16) |

MCA: Middle Cerebral Artery; ICA: Internal Carotid Artery; PCA: Posterior Cerebral Artery; LVO: Large Vessel Occlusion; EEG: Electroencephalography.

**Table S2: Summary of commonest EEG techniques**

| CHARACTERISTIC | (Number of studies providing this information) | BREAKDOWN  (Number of studies in each category) |
| --- | --- | --- |
| No. of electrodes | 30 | 1(3), 4(1), 6(1), 8(1), 16(7), 17(1), 18(1), 19(7), 20(2) 32(1), 62(1), 64(2), 19-128(1), 256(1) |
| Electrode material | 10 | Silver/Silver Chloride (8), Copper/Silver Chloride(2) |
| Reference electrode | 20 | Vertex (Cz) (4), Mastoid/Ear (A1/2) (10), Midline Frontal (Fz;FpZ) (2), Lead pair(1), Combination of sites (3) |
| Montage | 27 | Monopolar (19), Bipolar (6), Both (2) |
| No. of channels | 30 | 1(3), 4(1), 4 and 19(1), 8(1), 10(1), 14(1), 16(7), 18(1), 19(6), 20(1), 27(1), 62(1), 64(3), 19-128(1), 256(1), |
| Eye closure | 19 | Periods of both open and closed (6), Closed (11), Open(1), Controls closed (1) |
| Alertness | 19 | Conscious (1), Alert(4), Resting (7), Resting with vigilance control (7) |
| Bandpass filter - Hz | 17 | .01-100(3), 0.1-59(1), 0.16-70(1), 0.2-40(1), 0.3-30(2), 0.35-35(1), 0.5-30(3), 0.5-50(2), 0.5-70(1), 0.53-30(1), 1-50(1) |
| Lower filter – Hz | 9 | 0.16(1), 0.3(4), 0.5(2), 0.53(1), Sixth order 50(1) |
| Upper filter –Hz | 9 | 30(5), 50(1), 70(2), 76(1) |
| Impedance - kOhms | 21 | <5(10), <10(3), <=10(2), <15(1), <=20(1), 10-50 (1), <100(1), "Minimised" (2) |
| Sampling duration – seconds | 29 | 1(1), 2(1), 2.05(1), 4(1), 16(2), 22-60(1), 60(3), 62 (3), 80(1) 120(1), 128(1), 180(1), 240(1), 300(3), 300-600(1), 600(2), 1800(2), 1800-3600(1), 3072(2) |
| Sampling frequency | 10 | 100 Hz(1), 200Hz(1), 220Hz(1), 250Hz(2) 256 or 512 Hz(1), 500Hz(2), 1000 Hz(2) |
| No of EEGs | 31 | 1(22), 2(4), 3(1), <=6(1), 1 or more(1), biennial for the duration of the study(1), "Several"(1) |
| Artefact removal | 23 | Visual(8), Visual/Electro-oculogram(3), Visual/Electro-oculogram/Electro-myogram/Electro-cardiogram(1), Visual/Electro-oculogram/Digital(1),  Visual/Digital(1), Electro-oculogram(1), Electro-oculogram/minimised during recording(1), Digital(3), Digital/minimised during recording (1), Method not stated (3) |
| Transformation | 24 | Fast Fourier (15), Fast Fourier with Hamming/cosine window(8), Fast Fourier with Wavelet(1) |
